# Marked Gingival Overgrowth Protruding from the Oral Cavity Due to Sodium Valproate

**DOI:** 10.3390/diagnostics15020205

**Published:** 2025-01-17

**Authors:** Mami Uegami, Hiroaki Ito, Tadashi Shiohama

**Affiliations:** 1 Department of Pediatrics, Kameda Medical Center, 929 Higashi-cho, Kamogawa-shi 296-8602, Chiba, Japan; ito.hiroaki@kameda.jp; 2 Department of Pediatrics, Graduate School of Medicine, Chiba University, 1-8-1 Inohana, Chuo-ku, Chiba-shi 260-8670, Chiba, Japan; asuha_hare@yahoo.co.jp

**Keywords:** gingival overgrowth, sodium valproate, paroxysmal sympathetic hyperactivity

## Abstract

Drug-induced gingival overgrowth is associated with various systemic diseases, including epilepsy. Among antiepileptic medications, phenytoin is commonly reported to cause this condition. In contrast, sodium valproate (VPA), another widely used antiepileptic drug, rarely induces gingival overgrowth. This difference in side effects highlights the variability in drug-induced oral complications among different antiepileptic medications. This case study presents a patient who developed significant gingival overgrowth after using VPA for over 10 years. The study aims to identify VPA as the causative agent and observe changes during long-term administration and after dose reduction. Our findings demonstrate that even long-standing gingival overgrowth can improve rapidly following discontinuation of the causative medication, providing valuable insights for managing similar cases in the future.

**Figure 1:**
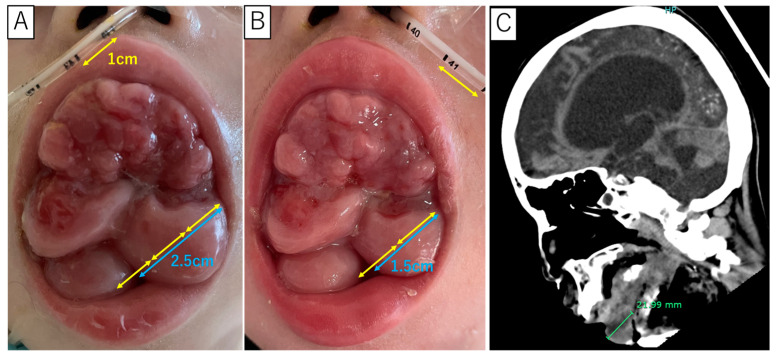
Gingiva overgrowth during VPA treatment (**A**) and one month after drug discontinuation (**B**). The computed tomography image depicts gingival overgrowth without overt abscess or tumor formation (**C**). Systemic diseases associated with drug-induced gingival overgrowth include hypertension, epilepsy, and autoimmune diseases. Among antiepileptic drugs, phenytoin is the most frequently reported to cause gingival overgrowth, although its incidence varies considerably [1,2]. VPA has also been reported to cause gingival overgrowth, but such cases are rare [3]. Treatment for drug-induced gingival overgrowth typically requires appropriate gingival care and medication modification, which can be challenging if seizures are not well controlled. This case study focuses on a patient who developed significant gingival overgrowth after using VPA for over 10 years. To our knowledge, there have been no previous reports on the long-term course of gingival overgrowth induced by VPA or its regression after dose reduction, making this case noteworthy. The study aims to identify VPA as the causative agent of gingival overgrowth and to observe changes during long-term administration and after dose reduction. A 12-year-old girl had nearly drowned in a bathtub when she was one year old. After being resuscitated, she suffered from severe long-term complications, including prolonged impaired consciousness. She required mechanical ventilation through a tracheostomy tube and received nutrition through a nasogastric tube. Despite having no family history of epilepsy, she began experiencing seizures at one year of age due to brain atrophy resulting from post-resuscitation encephalopathy. She was subsequently treated with VPA. Since then, she had several episodes of pneumonia and urinary tract infections. Gingival thickness and redness appeared on her anterior maxillary teeth at the age of four. They progressively extended to the entire maxillary dentition, causing protrusion from the oral cavity by age 10. The standard treatment for gingival overgrowth involves proper plaque management and discontinuing the suspected medication as the first choice [4]. However, considering the risk of worsening epilepsy, we did not discontinue the suspected medication, VPA. Due to communication challenges and mechanical ventilation, surgical options were deemed too risky. Head computed tomography (CT) (C) revealed no abscesses or caries inside the tumor-like gingival overgrowth, leading to the decision to follow up without surgical resection. Since the age of two years, she had experienced episodes of tachycardia, facial flushing, and myoclonus. She decreased SpO_2_ lasting for about three hours once or twice a month and continued to take VPA for presumed epileptic seizures. At the age of 12 years, these episodes were diagnosed as paroxysmal sympathetic hyperactivity (PSH) [5] instead of epileptic seizures because there was no EEG abnormality and no response to antiepileptic drugs such as midazolam (0.15 mg/kg/dose). Therefore, VPA was tapered off. One month after VPA reduction, the gingival overgrowth volume improved significantly (A, B), confirming the diagnosis of VPA-induced gingival overgrowth. Regarding the PSH episodes, starting a small dose of propranolol resulted in no episodes after discontinuing VPA. Unfortunately, the patient died of suspected sepsis three months later. Due to the risk of exacerbating epilepsy, discontinuing suspected drugs with a low causative frequency is challenging. Over the past 10 years of case reports, 7 out of 24 cases (29%) showed improvement in gingival overgrowth with just medication cessation and oral care, without the need for surgical treatment (Table S1) [4,6,7,8,9,10,11,12,13,14,15,16,17,18,19,20,21,22,23,24,25]. However, there have been no reported cases of improvement in gingival overgrowth after stopping medication following more than 10 years of long-term use. There have been no reports of gingival overgrowth due to VPA in the past 10 years. Our case demonstrates the potential for long-standing gingival overgrowth to regress rapidly after drug discontinuation.

## Supplementary Materials

The following supporting information can be downloaded at: https://www.mdpi.com/article/10.3390/diagnostics15020205/s1, Table S1: A review of the medications that cause gingival overgrowth and their treatment.
