# Supplementary material for: Marked Gingival Overgrowth Protruding from the Oral Cavity Due to Sodium Valproate
Source: Diagnostics (Basel). 2025 Jan 17;15(2):205. doi: 10.3390/diagnostics15020205 (PMC11765381; doi:10.3390/diagnostics15020205)
Supplement: Supplementary file 1 [file diagnostics-15-00205-s001.zip › diagnostics-3319353-supplementary.pdf]

Table S1
A review of the medications that cause gingival overgrowth and their treatment.

Author (year)	age, sex	Causative drug	Duration from starting	treatment
Nanda, T. et al. (2019) [6]	42 years old, man	Cyclosporine A, amlodipine	N.D.	Surgical intervention
Nivethitha, K. et al.(2020) [7]	21 years old, man	tacrolimus	two weeks	Surgical intervention
Morikawa, S. et al.(2020) [8]	66 years old, man	nifedipine, amlodipine	N.D.	Surgical intervention
Sharma, R. et al.(2020) [9]	21 years old, man	phenytoin	six months	Discontinue oral medication
Quenel,L. et al.(2020) [10]	56 years old, man	amlodipine	N.D.	Surgical intervention
Gandhi, M. Et al.(2020) [11]	12 years old, female	Cyclosporine A	five months	Surgical intervention
Fang, L. et al.(2021) [12]	58 years old, man<br>53 years old, female<br>59 years old, female	Cyclosporine A, nifedipine<br>nifedipine<br>nifedipine	ten years<br>ten years<br>eight years	Surgical intervention<br>Surgical intervention<br>Discontinue oral medication
Damdoum,M(2021) [13]	68 years old, female	amlodipine	five years	Surgical intervention
Tillmann, F.P. (2021) [14]	66 years old, female	Cyclosporine A	six months	Discontinue oral medication
James, J. et al. (2022) [15]	14 years old, man	levetiracetam	five days	Discontinue oral medication
Liu, Y. et al.(2022) [16]	42 years old, man<br>67 years old, female	Tacrolimus, Felodipine<br>amlodipine	one year<br>six years	Surgical intervention<br>Surgical intervention
Bhandari, S. et al.(2022) [17]	49 years old, female	amlodipine	one year	Surgical intervention
Kamei, H. et al.(2022) [18]	80 years old, female	amlodipine	N.D.	Surgical intervention
Okumus, O. F. (2022) [19]	56 years old, man	amlodipine	one year	Surgical intervention
Bakshi, S. S. et al. (2023) [20]	65 years old, female	amlodipine	two month	Discontinue oral medication
Dalal, R. et al. (2023) [4]	12 years old, man	Phenytoin	one year	Discontinue oral medication
Zisis, V. et al. (2023) [21]	38 years old, female	Cyclosporine A	N.D.	Discontinue oral medication
Tanoue, N. et al.(2023) [22]	58 years old, man	phenytoin<br>amlodipine	N.D.	Surgical intervention
Dhalla, N. et al. (2024) [23]	20 years old, female	phenytoin	four year	Surgical intervention
Alanija, L. et al.(2024) [24]	34 years old, female	amlodipine	N.D.	Surgical intervention
MihaiL.L.et al.(2024) [25]	71 years old, man	nifedipine	six months	Surgical intervention

Abbreviations : N.D., not described
